# Genome-wide association study and transcriptome analysis discover new genes for bacterial leaf blight resistance in rice (*Oryza sativa* L.)

**DOI:** 10.1186/s12870-021-03041-2

**Published:** 2021-06-03

**Authors:** Xinyue Shu, Aijun Wang, Bo Jiang, Yuqi Jiang, Xing Xiang, Xiaoqun Yi, Shuangcheng Li, Qiming Deng, Shiquan Wang, Jun Zhu, Yueyang Liang, Huainian Liu, Ting Zou, Lingxia Wang, Ping Li, Aiping Zheng

**Affiliations:** 1 College of Agronomy, Sichuan Agricultural University, Chengdu, China; 2 Rice Research Institute of Sichuan Agricultural University, Chengdu, China; 3 State Key Laboratory of Crop Gene Exploration and Utilization in Southwest China, Chengdu, China; 4 College of Life Science and Technology, Yangtz Normal University, Chongqing, China

**Keywords:** Rice, *Xanthomonas oryzae* pv. *oryzae*, GWAS, RNA-Seq, Resistance genes

## Abstract

**Background:**

Rice (*Oryza sativa*) bacterial leaf blight (BLB), caused by the hemibiotrophic *Xanthomonas oryzae* pv. *oryzae* (*Xoo*), is one of the most devastating diseases affecting the production of rice worldwide. The development and use of resistant rice varieties or genes is currently the most effective strategy to control BLB.

**Results:**

Here, we used 259 rice accessions, which are genotyped with 2 888 332 high-confidence single nucleotide polymorphisms (SNPs). Combining resistance variation data of 259 rice lines for two *Xoo* races observed in 2 years, we conducted a genome-wide association study (GWAS) to identify quantitative trait loci (QTL) conferring plant resistance against BLB. The expression levels of genes, which contains in GWAS results were also identified between the resistant and susceptible rice lines by transcriptome analysis at four time points after pathogen inoculation. From that 109 candidate resistance genes showing significant differential expression between resistant and susceptible rice lines were uncovered. Furthermore, the haplotype block structure analysis predicted 58 candidate genes for BLB resistance based on Chr. 7_707158 with a minimum *P*-value (–log 10 *P* = 9.72). Among them, two NLR protein-encoding genes, LOC_Os07g02560 and LOC_Os07g02570, exhibited significantly high expression in the resistant line, but had low expression in the susceptible line of rice.

**Conclusions:**

Together, our results reveal novel BLB resistance gene resources, and provide important genetic basis for BLB resistance breeding of rice crops.

**Supplementary Information:**

The online version contains supplementary material available at 10.1186/s12870-021-03041-2.

## Background

Rice (*Oryza sativa*) is providing approximately 20% dietary energy supply for world’s people [1]. However, rice production worldwide is severely threatened by bacterial leaf blight (BLB), a plant disease caused by *Xanthomonas oryzae* pv. *oryzae* (*Xoo*) [2]. *Xoo* is now prevalent in rice-growing areas of world, but due to its host-shifting capacity, this bacterium also threatens wheat production in both south America and Asia [3, 4]. Damage from this disease has led to rice production losses of 20%–30%, reaching devastating levels of up to 80%–90% in India and Philippines [5, 6]. Generally, this damage begins at the tillering stage, becoming more widespread as the incidence of disease increases with host plant growth peaking at the flowering stage [7]. The easiest way to prevent BLB is to apply chemical pesticides; however large-scale use of a variety of pesticides threatens the safety of rice food products. Additionally, because BLB spreads rapidly, such chemical control applied in a monsoon climate is ultimately unpractical, since once a BLB infestation occurs on a large scale, its effective control by pesticides is difficult if not impossible [8]. Therefore, developing and applying resistant rice cultivars is the most effective way to control this disease and ensure food security.

To date, more than 42 BLB resistance genes and hundreds of quantitative trait loci (QTLs) have been detected in rice plants. Among these genes, some were identified from wild species [9–11]. Other resistance genes or alleles have been found by mutating cultivated rice lines [12, 13]; e.g. *Xa1*, *Xa3/Xa26, Xa21*, *Xa23*, *Xa27*, *xa5*, *xa13*, *xa25*, and *xa41* [14–22]. Moreover, the complete spectrum of BLB R-genes reportedly consists of 16 genes: *xa5*, *xa8*, *xa9*, *xa13*, *xa15*, *xa19*, *xa20*, *xa24*, *xa25*, *xa26b*, *xa28*, *xa31*, *xa32*, *xa33*, *xa34*, and *xa42* [19, 22]. Importantly, BLB has high race specificity, and the rapid loss of BLB resistance in rice lines containing a single resistance gene remains a pressing problem for breeders. Combining multiple resistance genes or QTLs can contribute to broad spectrum and durable resistance to *Xoo* that is effective [23]. Therefore, detecting novel resistance genes and QTLs in many rice lines is imperative for successfully breeding rice capable of resisting BLB.

Most studies of BLB resistance have focused on a single resistant parent or bi-parental genetic mapping populations. To genetically map many agronomic traits and disease resistance loci in plants, genome-wide association study (GWAS) based on high-density single nucleotide polymorphisms (SNPs) and next-generation sequencing has been widely used [24, 25]. For example, Li et al. (2015) detected 97 loci associated with resistance to stripe rust in wheat via GWAS methods; and genes conferring resistance to *Verticillium dahlia* were identified in cotton by GWAS [26]. For rice, researchers have used GWAS to detect 27 loci related to rice blast resistance [27]. Yet due to its insufficient marker density and linkage disequilibrium (LD), GWAS does not provide an accurate target gene at a given locus. Transcriptome analyses can overcome this limitation by detecting and distinguishing the expression of candidate genes of different genotypes. Recently, Wen et al. [28] identified a set of candidate genes associated with white mold resistance in soya bean by combining transcriptome and GWAS approaches.

In this study, 259 rice lines were inoculated with two *Xoo* races P3 and P6, respectively, to evaluate their BLB resistance. We performed a GWAS of BLB resistance using 2 888 332 high-confidence SNPs (missing data < 20%; minor allele frequency [MAF] > 1%). Building on this, we explored candidate resistance genes by analyzing the transcriptomes of the most resistant rice line NSIC RC154 and the most susceptible line CT 9737–6-1-3P-M, at five post-infection time points (0, 12, 24, 48, and 72 h). Our results allowed us to detect candidate genes linked to BLB resistance in these rice lines. This should provide important gene resource for improving disease resistance breeding in rice.

## Results

### Genomic variation and population structure

**Fig. 1:**
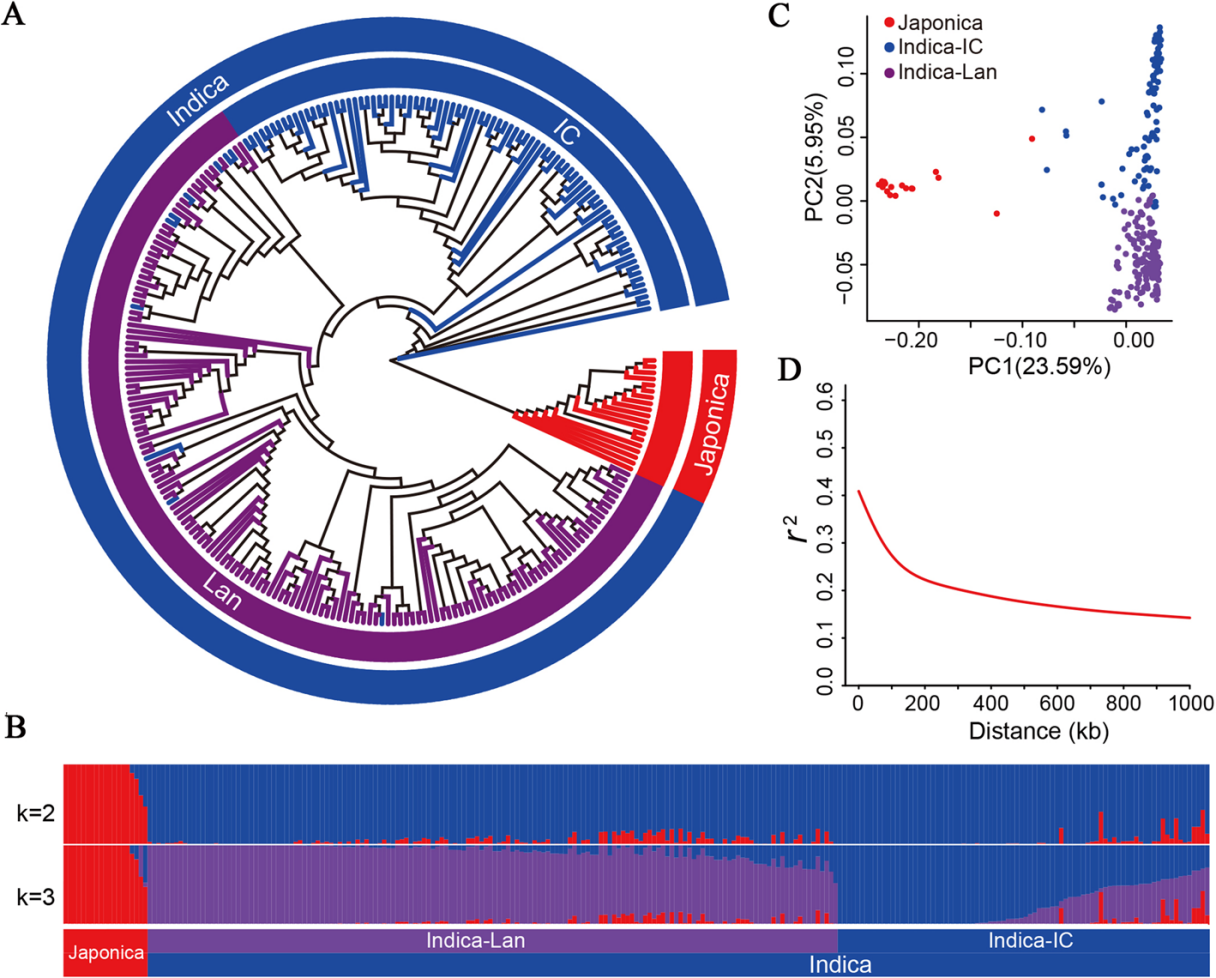
Population structure of 259 rice accessions. (**A**) Neighbor-joining phylogenetic tree generated using 2,888,332 high-quality SNPs; (**B**) Individual ancestry coefficients of 259 rice accessions determined using ADMIXTURE with the number of ancestry kinships (K) set to 2 or 3. Each accession is denoted by a vertical bar; the proportion of different colors in each bar indicates the proportion of genetic from each of the ancestral populations; (**C**) Principal component analysis (PCA) plot of 259 rice accessions. (**D**) Genome-wide average linkage disequilibrium (LD) decay rate of 259 rice accessions and three different subgroups

To search for BLB resistance genes, we sequenced 259 rice accessions using the Illumina Hi-Seq platform (Fig. 1A; Table S1). This generated 1.33 Tb of raw reads (Table S2), and 2 888 332 high-confidence SNPs (missing data < 20%; minor allele frequency [MAF] > 1%) were obtained after mapped onto the Nipponbare rice genome (Table S3). Among 2 888 332 high-confidence SNPs, there are 1 146 191, 555 884, 363 883, 464 911, and 318 546, SNPs were located in intergenic regions, exons, upstream regions, introns, and downstream regions, respectively (Table S3). In the coding sequences (CDS), the 16 416 stop-gain, 312 857 nonsynonymous, 2 850 splicing, and 1 085 stop-loss SNPs were found (Table S3). The number of SNPs among the 12 rice chromosomes ranged from 334 353 (Chr. 1) to 192 058 (Chr. 9), and the highest SNPs’ frequency were found on Chr. 8 (8.50 SNPs/kb) (Table S4). This SNP data set of 259 rice lines provides an abundant resource for use in the molecular improvement of BLB resistance in rice.

Population-structure and phylogenetic-tree analysis indicated that these 259 rice varieties contained three subgroups (K = 3): landrace indica cultivar (94), improved indica cultivar (146) and japonica (19) (Fig. 1B). According to the principal component analysis (PCA) of these 259 rice lines, the 29.54% genetic variation was explained on the first two PCs (Fig. 1C). These results indicated that the rice varieties used in this study harbor abundant genetic variation in the core germplasm of rice. The LD decay for 259 rice cultivars was estimated to be 194 kb (Fig. 1D), suggested the rice lines exhibited moderate LD [29].

### Phenotypic variation among rice cultivars

**Fig. 2:**
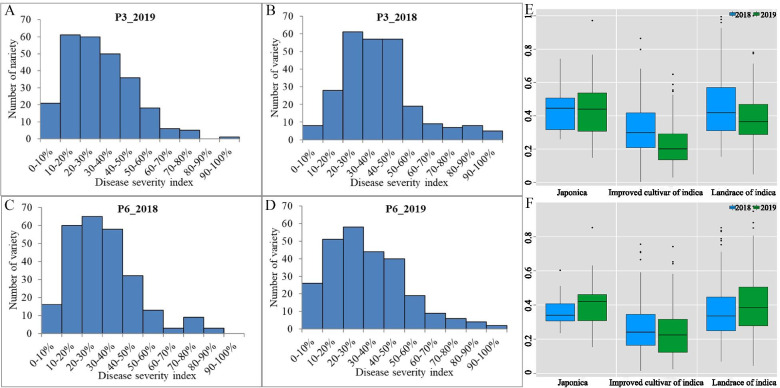
Susceptible and resistant reactions of 259 rice lines inoculated with two *Xanthomonas oryzae* pv. *oryzae* (*Xoo*) strains. (**A**, **B**) The frequency distribution of incidence rate of 259 rice lines after inoculation with *Xoo* P3 strain at 2 years; (**C**, **D**) The frequency distribution of incidence rate of 259 rice lines after inoculation with *Xoo* P6 strain at 2 years; (**E**) Histograms and box-plots showing the resistance phenotypic data of three rice subgroup to *Xoo* P3 strain; (**F**) Histograms and box-plots showing the resistance phenotypic data of three rice subgroup to *Xoo* P6 strain

All 259 rice lines were inoculated with two *Xoo* races (i.e., P3 and P6) at the booting stages, and the resistant phenotypes to each were investigated over 2 years. For the P3 *Xoo* race, the BLB disease severity index (DSI) in all lines ranged from 0.26% to 99.46% in 2018 (average = 37.94%) and from 2.82% to 100% in 2019 (average = 30.34%). For P6, the corresponding BLB DSI values were 1.46–85.32% (2018; average = 30.92%) and 2.49–97.84% (2019; average = 31.92%) (Fig. 2A-D; Table S1). The wide range of BLB DSI values observed in the different rice lines (a 16- and 12-fold difference, respectively, for the two *Xoo* races) demonstrated substantial genotypic variability is associated with rice resistance to BLB. Additionally, the BLB incidence rate for the two races was normal (Fig. 2A-D). Analysis of variance (ANOVA) of BLB DSI for both races revealed significant differences among genotypes, implying the presence of dominant loci conferring BLB resistance. Crucially, the level of BLB resistance was highest among the improved cultivars and lowest among the landraces (Fig. 2E, F). This latter result indicated that artificial selection has been successful in rice breeding applications.

### GWAS for bacterial leaf blight resistance

**Fig. 3:**
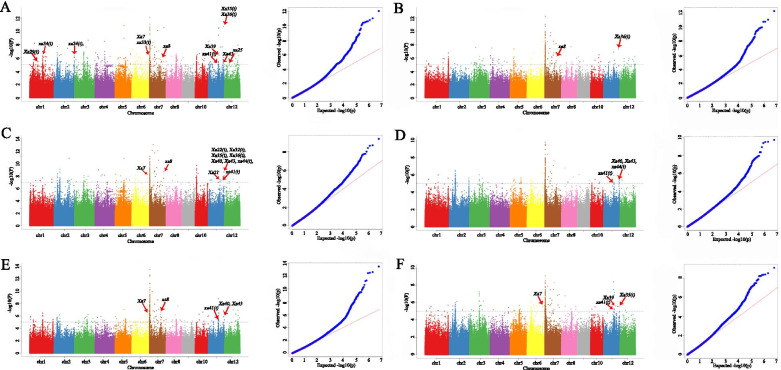
Manhattan and quantile–quantile plots resulting from the genome-wide association study (GWAS) for bacterial leaf blight (BLB) resistance in rice. (**A**, **B**, **C**) GWAS for BLB resistance in (**A**) P3_2018, (**B**) P3_2019 and (**C**) Best linear unbiased prediction (BLUP) values of P3 at 2 years. (**D**, **E**, **F**) GWAS for BLB resistance in (**D**) P6_2018, (**E**) P6_2019 and (**F**) Best linear unbiased prediction (BLUP) values of P6 at 2 years. The x-axis shows the single nucleotide polymorphism (SNPs) along each chromosome; the y-axis is the –log_10_P for the association

Based on the 2 888 332 high-confidence SNPs, the GWAS of two resistance traits (each *Xoo* race was presumed to select for different plant trait) was conducted by a mixed linear model (MLM). For the GWAS analysis, phenotypic data of these two traits (in 2 years) and their best linear unbiased prediction (BLUP) values of each trait per year were used. A total of 196 and 164 SNP loci were significantly associated with resistance to P3 and P6, respectively (− log_10_P ≥ 5) (Fig. 3; Tables S5, S6, S7, S8, S9 and S10). Intriguingly, 63 SNP loci of them were common to all traits. Next, the GWAS was applied to the selected 240 varieties of indica rice. Similarly, the phenotypic data of the two traits and their yearly BLUP values were used this analysis. Among significant association SNPs, when compared with results of the GWAS implemented for all 259 rice varieties, the strongest signals was also identified on Chr.7 (Figure S1). However, some known loci did not appear in the GWAS results of 240 indica rice varieties (Fig. 3 and Figure S1). Accordingly, the 19 japonica rice varieties can contribute to the BLB GWAS, so the GWAS results of all 259 rice varieties were investigated further. Candidate genes were detected within 200 kb upstream and downstream of the significant associated SNP, according to the LD decay of the rice genome (Fig. 1D). A total of 2 081 genes (significant for 2 years and BLUP values) were detected for P3, and likewise 1 297 genes were significantly detected for P6. Of those, 954 genes were common to both resistance traits (Tables S5, S6, S7, S8, S9 and S10). The number of these genes was higher on Chr. 7 and Chr. 11, when compared with the other rice chromosomes (Table S5, S6, S7, S8, S9 and S10).

We summarized 41 previously reported fine-mapped QTLs or genes related to BLB resistance using Oryzabase database (http://www.shigen.nig.ac.jp/rice/oryzabase/gene/list). To further confirm these significant genes or SNPs found associated with BLB, the results were compared with those of 41 previously reported fine-mapped QTLs or genes. Of the loci detected in the P3 strain, the sixteen previously QTLs fine-mapped QTLs or genes were identified (Fig. 3A-C). For the loci that were detected in the P6 strain, seven SNPs were adjacent to previously reported QTL or genes (Fig. 3D-F). This confirmed that our results were reliable, has a strong potential for more deeply exploring novel resistance genes in rice.

### Comparison of the transcriptomes of resistant NSIC RC154 and susceptible CT 9737–6-1-3P-M lines of rice

Because of the strong population structure and large extent of LD in rice, GWAS-identified loci often fall within gene deserts or in regions with many equally plausible causative genes. This makes it difficult to robustly identify functional genes. Combining the results of GWAS with RNA-seq data has been used to detect the function genes.

Furthermore, previous reports also indicated that genes that were present different expression levels in different resistance lines are most likely to be association with disease resistance [28].

Among the 259 lines tested in the 2-year field trial, NSIC RC154 (DSI = 6.01%) and CT 9737–6-1-3P-M (DSI = 100%) emerged as the most resistant and susceptible lines, respectively. To further identify those genes related to BLB resistance, we performed a transcriptome analysis on leaves from both lines to analyze the levels of gene expression at 12, 24, 48, and 72 h post-inoculation (hpi) with *Xoo*. Overall, we respectively obtained 2, 080, 282, 498 clean reads (Table S11), and the mapped reads value was 91.89–94.29% and 91.81%-94.37% for NSIC RC154 and CT 9737–6-1-3P-M, respectively (Table S11). The resistant line NSIC RC154 and the susceptible line CT 9737–6-1-3P-M exhibited a substantially different response to the *Xoo* inoculation; the number of differentially expressed genes (DEGs) in NSIC RC154 (11 674; 3 167 up-regulated and 8 507 down-regulated) (Table S12) exceeded those in CT 9737–6-1-3P-M (11 436; 2929 up-regulated, 8 254 down-regulated) (Table S13). Among these DEGs, although 8 851 DEGs of them were common by the two rice varieties at different time points, 2 817 DEGs were only found in the resistance line NSIC RC154 (Table S14). Therefore, those latter genes may figure prominently in conferring resistance to BLB. At earlier stages (12 h and 24 h) of inoculation, 9 023 DEGs (2 477 up-regulated, and 6 546 down-regulated) were detected in the resistant line NSIC RC154, compared with 8 040 DEGs (2 201 up-regulated, and 5 839 down-regulated) in the susceptible line CT 9737–6-1-3P-M. When challenged with the pathogen, NSIC RC154 harbored more DEGs than did CT 9737–6-1-3P-M at the early stages. This suggested that NSIC RC154 reacts more strongly than CT 9737–6-1-3P-M to pathogen attack and invasion. We presumed that NSIC RC154 might have numerous resistance-related genes that are primed for a quick response to pathogen infection.

### Expression analysis of the GWAS-identified genes

**Fig. 4:**
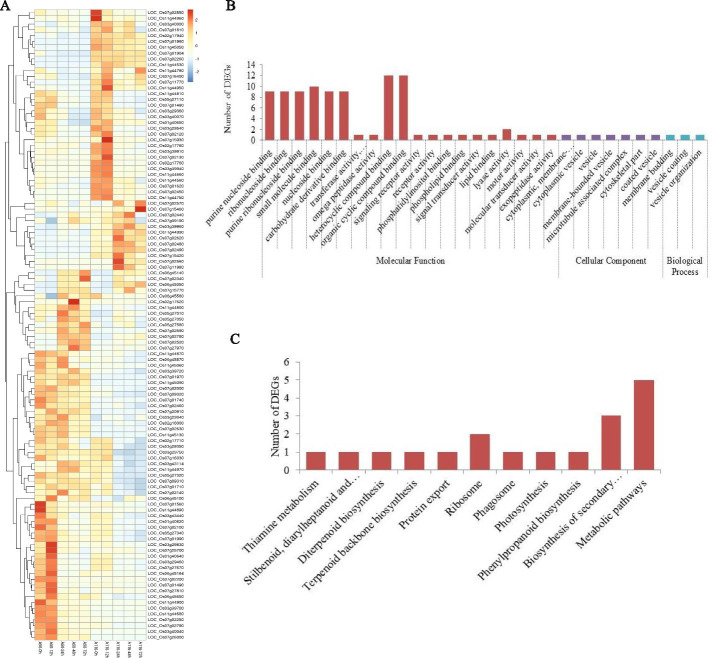
The candidate genes were detected via GWAS and different expression genes (DEGs) data set. (**A**) Heat map for expression patterns of the 109 candidate genes at 12, 24, 48, and 72 h post inoculation with Xoo strain P6 in R genotype (NSIC RC154) and S genotype (CT 9737–6-1-3P-M); (**B**) GO classification of candidate genes. Gene Ontology terms are classified into three main categories: biological process, cellular component, and molecular function; (**C**) KEGG annotation of putative proteins. The y-axis indicates the name of the KEGG metabolic pathway. The x-axis indicates the percentage of the number of unigenes annotated to the pathway out of the total number of unigenes annotated

Among the 954 genes found significantly associated with resistance against the two *Xoo* races, 161 (36 up-regulated, 125 down-regulated) were DEGs in the resistant rice line NSIC RC154, and 197 (64 up-regulated, 140 down-regulated) were DEGs in the susceptible rice line CT 9737–6-1-3P-M. Furthermore, a set of 109 DEGs that underwent significant differential expression between resistant and susceptible rice lines was detected (Fig. 4A). Gene Ontology (GO) analysis revealed a stark enrichment of DEGs in several functional categories, namely signal transducer activity, purine nucleoside binding, receptor activity, motor activity, and molecular transducer activity (Fig. 4B). The KEGG (Kyoto Encyclopedia of Genes and Genomes) annotations indicated that the pathways enriched with these DEGs were closely related to phenylpropanoid biosynthesis, biosynthesis of secondary metabolites, photosynthesis, diterpenoid biosynthesis, as well as the biosynthesis of stilbenoid, diarylheptanoid and gingerol (Fig. 4C). Elucidating the detailed mechanisms of BLB resistance based on these results is difficultly; however, these DEGs were great importance as they most likely as candidate genes for enhancing resistance to BLB.

Furthermore, among those 109 DEGs with significant differential expression levels between resistant and susceptible rice lines, nearly half (45) were expressed more in the resistant than susceptible line (Fig. 4A). These 45 genes contain a large number of defense-associated protein encoded genes, such as the NBS-LRR disease resistance protein encoding the genes *LOC_Os11g44960*, *LOC_Os11g45050*, *LOC_Os07g02570*, *LOC_Os11g44990*, *LOC_Os07g02620*, and *LOC_Os07g02560*. It is known that the NBS (nucleotide-binding state)-LRR (Leucine-rich repeat) disease resistance protein is critically involved in plant-pathogen interactions, and its NBS play a key role in regulate activity of this protein [30]. Perhaps more importantly, of the six NBS-LRR disease resistance protein-encoding genes, *LOC_Os07g02570*, *LOC_Os11g44990*, *LOC_Os07g02620*, and *LOC_Os07g02560* displayed induced up-regulation in the resistant line at 24 hpi, whereas no significant changes in those four genes were detectable in the susceptible line after the *Xoo* inoculation. Reactive oxygen species (ROS) is important in plants by triggering plant basal defense responses [31]. The peroxidase precursor-encoding gene, *LOC_Os07g02440*, was up-regulated in the resistant line at 24 hpi, but not induced in the susceptible line at all time points tested (Fig. 4A). The expression of this gene implies that ROS could be crucially involved in marshaling defense against the *Xoo* pathogen early in its infection of rice.

Moreover, the natural-resistance-associated macrophage protein (NRAMP)-encoding gene *LOC_Os07g15460*, the glycosyl hydrolase family 3 protein-encoding gene *LOC_Os11g44950*, and the cytochrome P450-encoding gene *LOC_Os02g17760* all underwent significantly higher expression in the resistant line compared with the susceptible line. According to other research, iron is a key element for most living organisms, and pathogens are likely to compete with their hosts for its acquisition [32]. The bacterial plant pathogen *Dickeya dadantii* depends strongly on its siderophore-mediated iron uptake system for systemic disease progression on several host plants, including *Arabidopsis thaliana* [33]. In rice plants, several metal ions such as Zn^2+^, Mn^2+^, Fe^2+^, and Cd^2+^ are transported via NRAMP transporter proteins [34]. In our study, the NRAMP-encoding gene *LOC_Os07g15460* was found up-regulated in the resistant line at 72 hpi, yet it underwent no significant expression change in the susceptible line. This gene may thus contribute to resistance to BLB via iron transfer. Furthermore, three protein kinase-encoding genes (*LOC_Os11g44660*, *LOC_Os11g44560*, and *LOC_Os07g02450*) were down-regulated in the resistant line by the *Xoo* inoculation, but their expression levels were not induced in the susceptible line’s transcriptome at any time point examined. Further research should try to functionally validate effects of these genes, which is needed to reveal the molecular mechanisms of complex BLB resistance traits in rice.

### Expression validation of candidate genes by qRT-PCR

**Fig. 5:**
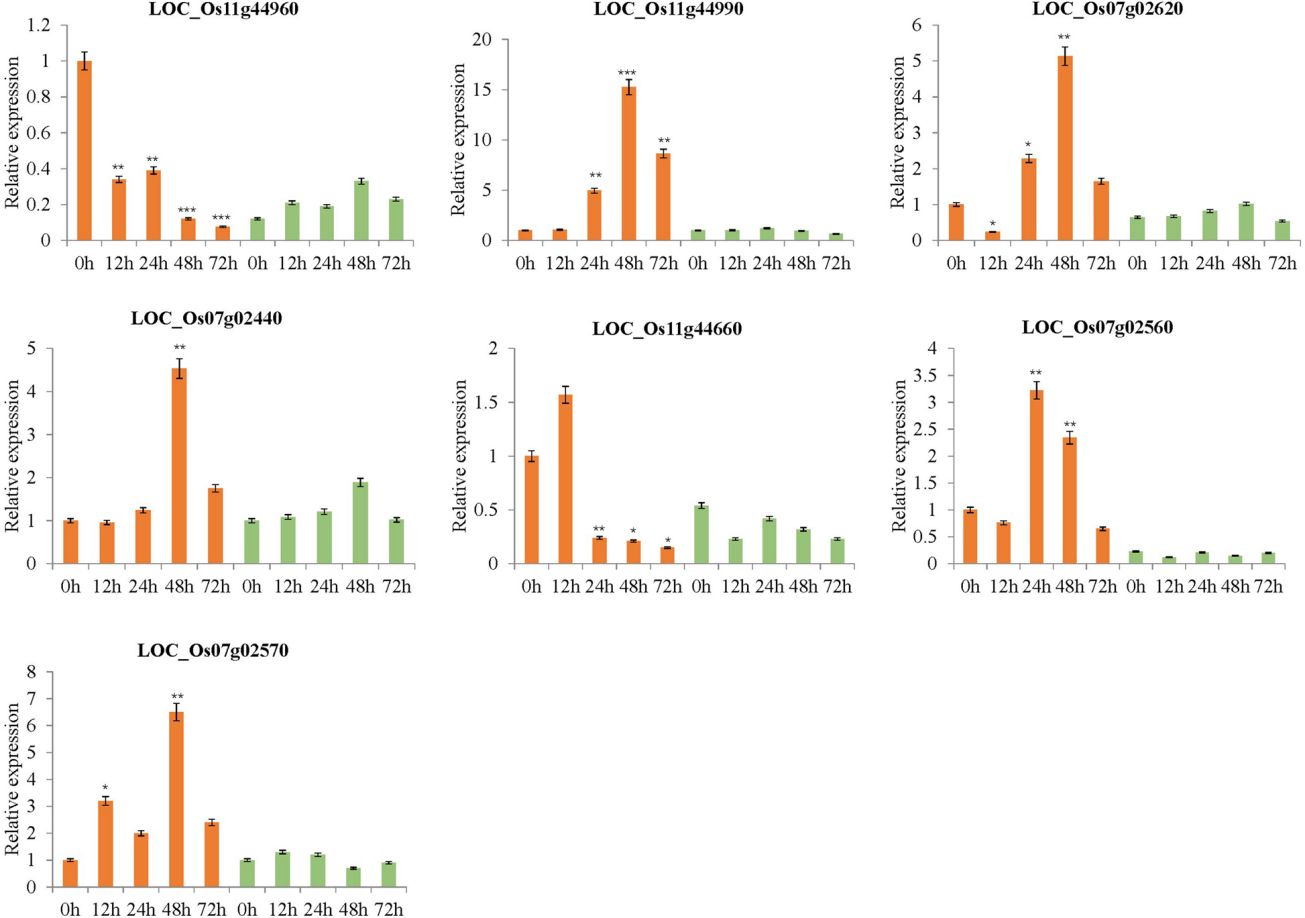
Haplotype analysis of the peak associated with the gene on chromosome 7. (**A**) Manhattan plots of loci on chromosome 07 associated with BLB incidence. LD heat map (bottom) reflected that associated SNP localized in a haploid between the red dashed lines; (**B**) Expression of two NBS-LRR genes LOC_Os07g02560 and LOC_Os07g02570 in the NBS-LRR gene cluster in resistant and susceptible rice lines (Transcriptome data); (**C**) Expression analysis of LOC_Os07g02560 and LOC_Os07g02570 in six resistant and six susceptible varieties at 24 h post inoculation (hpi) of *Xoo* by qRT–PCR

The seven candidate genes were selected for verify the RNA-Seq data. These consisted of six encoding NBS-LRR disease resistance protein (*LOC_Os11g44960*, *LOC_Os07g02560*, *LOC_Os07g02570*, *LOC_Os11g44990*, and *LOC_Os07g02440*) and one protein kinase gene (*LOC_Os11g44660*) (Fig. 5). The threshold cycle (Ct) values of each gene were normalized relative to those of the UBQ gene (internal control). The relative expression levels of these genes were detected using qRT-PCR method and compared with transcriptome results. The results indicated that these genes expressed differently in the resistance line after the *Xoo* P6 strain inoculation, in way that was consistent with the RNA-Seq data (Fig. 5).

### Identification of new BLB resistance genes by haplotype and expression analyses

**Fig. 6:**
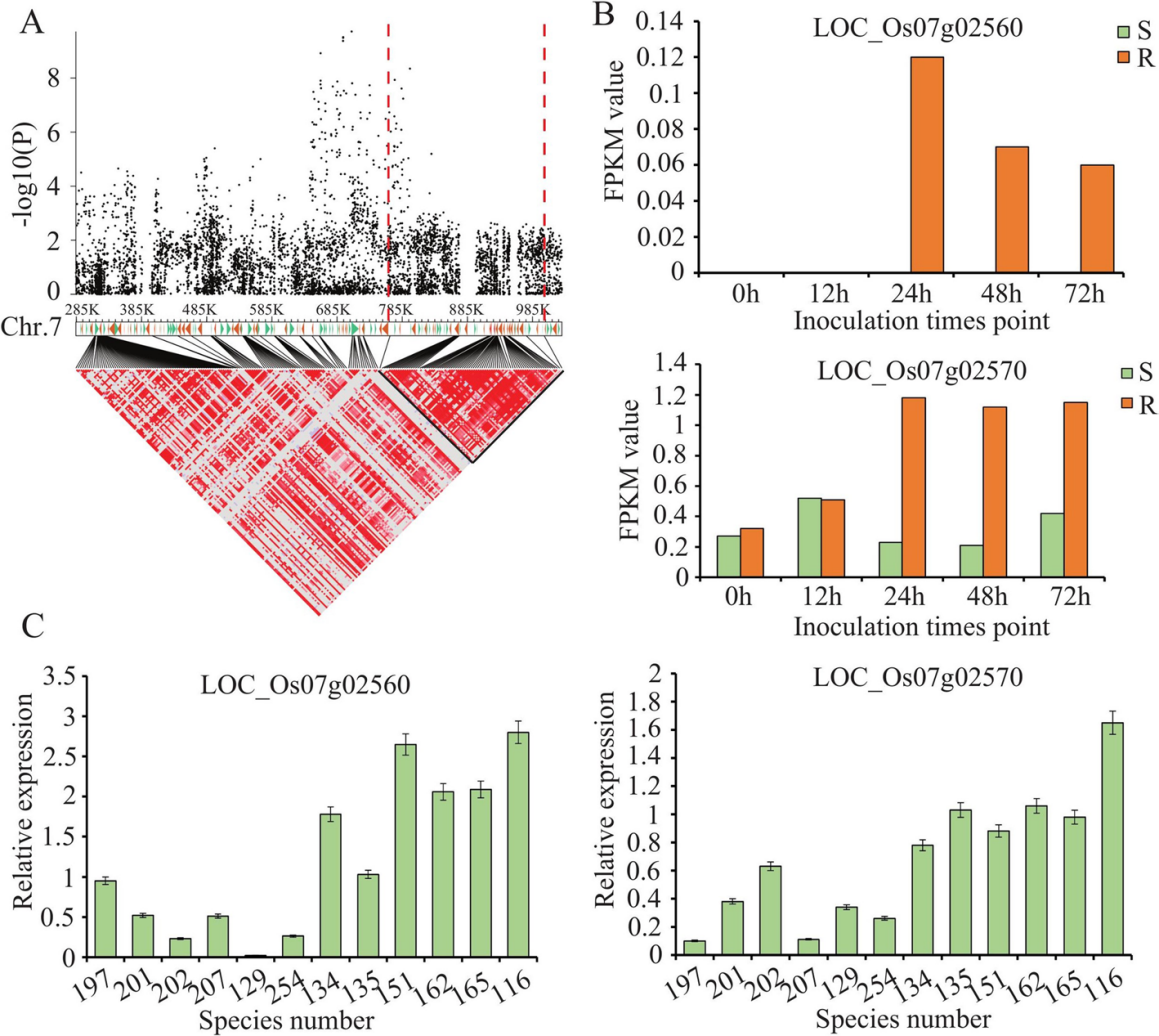
Verification of seven candidate resistant genes expression in resistant and susceptible rice lines at different infection time points by qRT-PCR. Error bars represent standard errors from four biological replicates (*, *P* < 0.05; **, *P* < 0.01; ***P < 0.001)

In this study, the strongest signals identified on Chr.7 were novel (Fig. 3), and the region of SNPs located on Chr.7 contained many genes that encode NBS-LRR resistance proteins. The candidate region was estimated using pairwise LD correlations. We focused on the locus mapped from 0.747 to 0.981 Mb with 58 candidate genes (Fig. 6A). Among these genes, the transcriptome data uncovered two NLR protein-encoding genes, *LOC_Os07g02560* and *LOC_Os07g02570*, which went significantly high expression in the resistant line but had low expression in the susceptible line of rice (Fig. 6B). To confirm the functioning of *LOC_Os07g02560* and *LOC_Os07g02570* in BLB resistance, six resistant and six susceptible varieties were selected to examine the expression levels of *LOC_Os07g02560* and *LOC_Os07g02570* after inoculation with *Xoo* P6. Expression of these two genes was higher in the resistant than susceptible varieties at 24 hpi (Fig. 6C). We also analyzed the sequences difference of these two candidate genes in resistant (NSIC RC154) and susceptible (CT 9737–6-1-3P-M) varieties. We found four SNPs (Chr.7_ 922,336, T-C; Chr.7_ 922,460, T-C; Chr.7_ 922,462, A-G; Chr.7_ 922,694, G-A) in upstream of LOC_Os07g02560, and six SNPs (Chr.7_ 920,121, C-A; Chr.7_ 920,184, T-C; Chr.7_ 920,534, G-A; Chr.7_ 920,762, G-A; Chr.7_ 920,766, C-T; Chr.7_ 920,861, T-G) were contained in coding region. For LOC_Os07g02570, two SNPs (Chr.7_927311, G-A; and Chr.7_928738, A-G) in upstream, and two SNPs (Chr.7_925408, A-G; and Chr.7_926479, A-G) in coding region were detected. These results indicated those two genes are significantly associated with resistance to BLB, and so they may be promising candidate resistance genes for this disease.

## Discussion

For 259 rice accessions, we evaluated their resistance levels to BLB over 2 years. The results showed that not all rice sub-populations were equally resistant. Moreover, among the resistant levels of the 259 rice lines, there was a remarkable disparity between the two *Xoo* pathogen races tested, which indicated that the R genes with specific resistance were only carried by certain rice varieties. This finding is consistent with those of a smaller study by Zhang et al. [35], in which the level of indica rice resistance differed significantly among six *Xoo* races, with the latter divided into three groups based on the lesions’ size (length values). Furthermore, the resistance level of each line could be inferred from their incidence rate: < 10% distinguished the resistant lines. Among our 259 rice accessions, six lines (IR 10M126, NSIC RC154, CT 16,658–5-2-3SR-2–1-MMP, CT 15,765–12-1–4-2–1-M, CT 15,765–13-3–8-3–3-M, IR61009-37–2-1–2, and HHZ 12-DT 10-SAL 1-DT 1) had considerably high and stable resistance to both *Xoo* races. These results suggest the possibility of selecting appropriate materials for accelerating BLB-resistance breeding and the genetic study of rice.

GWAS is an important approach for detecting the function genes of complex traits. It has been used to detect new genes associated with resistance to diseases and important agronomic traits in plants. In maize, for instance, Li et al. [36] used GWAS to illuminate the role of *ZmFBL41*, which encodes an F-box protein, in that corn plant’s resistance to sheath blight. In this study, we identified 63 BLB resistance loci, containing 954 significantly associated genes, though a GWAS of 2,888,332 high-confidence SNPs. The five significant SNPs found, namely Chr. 12_17579641, Chr. 11_28745675, Chr. 11_21379864, Chr. 08_27163888, and Chr. 11_17915331 were respectively located near the cloned R genes *xa25* [37], *Xa26* [14], *Xa21* [38], *Xa23* [39], *Xa33* [40], *xa13* [16], and *Os-11N3* [41] (Fig. 3). These results indicate that a relatively high resolution of GWAS is attainable when using a relatively large population, which considerably strengthened the investigation of genetic diversity and generation of a high-density SNP map for rice. By successfully combining the GWAS with transcriptome data to discover BLB resistance candidate genes in rice, novel loci were identified on chromosomes 7 (Chr.7_707158) that were significantly associated with rice resistance to both P3 and P6. Our data provide important information for future gene function studies of BLB resistance. It is anticipated these findings can serve as a robust reference for function gene discover on complex traits in rice and other plant species.

Compared with the susceptible line CT 9737–6-1-3P-M, the resistant line NSIC RC154 had more up-regulated genes at early time points following the pathogen inoculation. Furthermore, in combining the GWAS and transcriptome data, 109 significant associations with BLB-resistance DEGs were obtained. After assigning these genes to KEGG functional annotation, the pathways of phenylpropanoid biosynthesis, biosynthesis of secondary metabolites, photosynthesis, diterpenoid biosynthesis, and stilbenoid, diarylheptanoid, and gingerol biosynthesis were found to be all enriched. Phenylpropanoid compounds play key roles in plant defense, ranging from constitutive or inducible physical and chemical barriers against pathogenic infections, to acting as signal molecules involved in local and systemic signaling for the induction of one or more defense genes [42]. The enriched phenylpropanoid biosynthesis suggests that secondary defense metabolites figure prominently in early resistance to BLB in rice. Other pathways, however, such as diterpenoid biosynthesis and photosynthesis, likely also participated in plant disease resistance. For example, *Oscyp71Z2* governs BLB resistance by regulating the biosynthesis of diterpenoid phytoalexin [43]. Our results suggest that rice resistance to BLB is a rather complex trait, in that it depends on a well-coordinated and activated network of multiple defense pathways.

Many BLB resistance genes have been searched for and applied to rice breeding [44]. Although some *Xoo* resistance genes are now known, most are specific resistance genes; e.g., *xa25*, *xa26*, and *Xa1* [30, 45, 46]. Therefore, given the diminished plant resistance to BLB caused by evolving *Xoo* populations, it is imperative we find new genes conferring resistance traits and combine them with known resistance genes to develop durable and sustainable resistant lines of rice. In this study, multiple NLR genes were found localized at the Chr.7_707158 locus, where several NBS-LRR genes are also clustered. Through the haplotype and expression analyses, an NBS-LRR gene cluster (*LOC_Os07g02560* and *LOC_Os07g02570*) that confers broad-spectrum resistance in rice to both *Xoo* races was uncovered. But whether all these mutations in fact drove the loss of plant resistance to *Xoo* requires further careful investigation. Furthermore, plant genomes often will encode several hundred NLR proteins that are involved in defense responses, some of which occur in clusters at specific loci following gene duplication and amplification events. Previous reports indicate that genetically linked NLR genes may act together to recognize a pathogen’s avirulent effectors, such as *RPS4*/*RPS1*, *RGA4*/*RGA5*, and *Pikp-1*/*Pikp-2*. In such pairs, one gene functions as a “sensor” that perceives pathogen effectors while the other is a “helper” required to activate immune signaling [47–49]. Similarly, *LOC_Os07g02560* and *LOC_Os07g02570* were co-localized in an LD block. Yet whether these two NBS-LRR genes in the R-cluster are subject to the same regulatory mechanism as *RPS4*/*RPS1*, *RGA4*/*RGA5* or *Pikp-1*/*Pikp-2* is unknown and merits further study.

## Conclusions

We integrated the GWAS and transcriptome results of our study to provide some new, useful gene resources against bacterial blight in rice. Two candidate genes *LOC_Os07g02560* and *LOC_Os07g02570*, were thus obtained. These findings should provide reliable targets for assessing candidate genes for use in the breeding of BLB resistance. More work remains to be done, however, to verify which additional genes underpin resistance to BLB in rice.

## Methods

### Plant materials and phenotypic evaluation

A total of 259 rice lines were used in this study. These varieties were collected from different country, including Senegal, China, Malaysia, Brazil, Colombia, and Mexico. Of these, 146 lines were provided by the International Rice Research Institute (IRRI) while the other 113 had been preserved by the Rice Research Institute of Sichuan Agriculture University, China. Information of 259 rice lines can be found in Table S1. To evaluate BLB resistance, the seeds of all 259 rice varieties were sown in a greenhouse. Then transplant to an experimental field (at Sichuan Agriculture University) after 30-day-old, with 10 plants per row.

We used two representative *Xoo* strains P3 and P6 to artificially inoculate plants. The strains were cultivated separately on peptone dextrose agar (PDA) medium for 2 days at 30 °C; each *Xoo* race was suspending using sterile water at a concentration of 10^8^ cells ml^−1^ as inoculum. At the rice tillering stage, 15 of the uppermost leaves of each rice variety were infected with the two *Xoo* races, using the leaf-clipping method [50]. Lesion lengths were measured at 14 days after inoculation, when lesions were easily visible. A BLB disease score was recorded for each line, as the lesion length divided by the leaf length. The average disease score of each variety was calculated based on 15 individual leafs. Data were processed with Microsoft Excel 2010. Statistical analysis of BLB scores among different rice varieties or sub-populations was done using ANOVA followed by Dunnett’s multi-comparison test in SPSS v16.0 (IBM Corp., Armonk, USA).

### DNA extraction and sequencing

Young leaves of 21-day-old seedlings of each rice variety were sampled to extract their genomic DNA. The cetyl trimethyl ammonium bromide (CTAB) method was used to extract genomic DNA [51]. The purity and concentration of DNA was determined, respectively, through a NanoPhotometer spectrophotometer (IMPLEN, CA, USA) and a Qubit DNA Assay Kit with a Qubit 2.0 Fluorometer (Life Technologies, CA, USA). The DNA samples of all 259 rice varieties were first fragmented by sonication to 350 bp fragments. These DNA fragments were sequencing after end-polished, A-tailed, and ligated to full-length adapters. Next, the raw sequences having a 150-bp read length were obtained. Among raw data, those reads that contained adapter sequence stretches of –Ns, or had low quality scores were deleted. The high quality paired-end reads were mapped to the Nipponbare rice genome (ftp://ftp.ensemblgenomes.org/pub/plants/release_36/fasta/oryza_indica/dna/) by Burrows-Wheeler Aligner software tool with the command “mem -t 4 -k 32 –M” [52]. After their alignment, genomic variants in GVCF format for each accession were identified by the Haplotype Caller module and GVCF model in, Genome Analysis Toolkit (GATK) software [53]. All the GVCF files were then merged together. A raw genotype file was then filtered by these parameters: depth for each individual ≥ 4; minor allele frequency (MAF) ≥ 0.01, genotype quality for each individual ≥ 4; and; a miss rate ≤ 0.2. In this way, a total of 2 888 332 SNPs were obtains, and further annotated using ANNOVAR software (v2013-05–20) [42]. These SNPs were divided into five groups according to their annotations: CDS SNPs, upstream SNPs (those positioned within 1 kb of the transcription start site), intergenic SNPs, downstream SNPs (located within 1 kb of the transcription stop site), and intron SNPs.

### PCA, population structure, and LD analysis

The neighbor-joining (NJ) tree was built through the *P*-distance, by using the 2,888,332 SNPs in the Tree Best software (v1.9.2) and 1000 bootstrap replications [54]. The population structure of 259 rice lines was identified by the program ADMIXTURE (v1.23) [55], with a K-value ranging from 2 to 3. A principal component analysis (PCA) was then carried out using GCTA software [56]. To do this, the genetic relationship matrix was first calculated through the parameter “–make-grm”; the top three principal components were obtained by the parameter “–pca3”. To identify the LD of this rice population, the squared Pearson correlation coefficient (r^2^) between pairwise SNPs was calculated through the “Pop-LD-decay” software tool [57], whose program parameters were set to “-MaxDist 1000 kb-MAF 0.05 -Miss 0.1”. The average *r*^2^ value was calculated for pairwise markers in a 1-kb window, and these values averaged across the whole rice genome.

### Estimation of breeding value

The breeding values were calculated by BLUP (best linear unbiased predictor), using the “lme4” package in the R computing platform (v. 3.2.2) [58], as follows:

$$Y = \mu + Line + Loc + \left( {Line \times Loc} \right) + \left( {Rep \times Loc} \right) + \varepsilon$$

where, the *Y*, *μ*, *Line*, and *Loc* are respectively the phenotype, intercept, variety effects, and environmental effects. The *Rep* is the number of replications, and ε indicated the random effects; the *Line* × *Loc* term denotes the interaction between variety and environment, while *Rep* × *Loc* is the interaction between replication and environment.

### GWAS analysis

Only those SNPs with a sequencing depth ≥ 4, missing rate < 0.2 and MAF ≥ 0.01 were used in the GWAS, with the latter analyzed using the EMMAX (beta version) software package [59]. The matrix of pairwise genetic distances, which calculated using EMMAX, formed the variance–covariance matrix of random effects.

### Transcriptome analysis

To further identify candidate resistance genes positioned around the GWAS-identified loci, the resistant rice variety, NSIC RC154, and the susceptible variety CT 9737–6-1-3P-M (both confirmed) were grown and inoculated with the more virulent *Xoo* race P6 in a greenhouse by the leaf-clipping method [50]. From each rice variety, leaves sample were obtained at 12, 24, 48, and 72 hpi, respectively, and each treatment has three replicates. Control samples of non-inoculated, fresh leaves of seedlings at 12 h were also collected. Place all leaf samples in liquid nitrogen and stored at -80℃ for their RNA isolation. Total RNA was isolated with the Plant Total RNA Isolation Kit (Sangon Biotech, Shanghai, China), according to the manufacturer’s instructions. We used the NEBNext Ultra™ RNA Library Prep Kit for Illumina (NEB, USA) for RNA-Seq libraries construction. The Illumina Hi-Seq platform was used sequencing, and 125-bp paired-end reads were generated. Among raw data, the reads having a low quality score and those containing adaptor sequences and stretches of -Ns were removed. An index of the Nipponbare rice reference genome was built using Bowtie v2.2.3, to which the above paired-end reads were aligned using TopHat v2.0.12 [60–62]. To count the number of reads mapped to each gene, HTSeq v0.6.1 software was used [63]. The expression value of each gene was present based on FPKM (fragments per kilobase of transcript sequence per million) that calculated using Cuffdiff software (v2.2.1).

The differential expression analysis of two treatments (each treatment contains three biological replicates) was carried out in R, using the “DESeq” package (v1.18.0) [64]. Differential expression levels of gene in the two treatments sample comparisons were determined based on the negative binomial distribution. Benjamini and Hochberg’s approach was used to adjust *P*-values for controlling the false discovery rate (FDR). Genes with the |log twofold change |> 1 and adjusted *P*-values of < 0.05 were designated as differentially expressed [65].

### Haplotype analyses

Haplotype blocks were distinguished by using the confidence interval method [66], and Haploview software [67]. For this, the Hardy–Weinberg *P*-value cut-off was set to 0.001, with a MAF of 0.05.

### qRT-PCR

Relative expression levels of seven candidate genes were investigated in rice plants by qRT-PCR. Total RNA extraction and reverse transcription were performed as described previously. The PCR reactions were using 20µL volume, which contained cDNA template 3 µL and forward and reverse gene-specific primers 0.8 µL, respectively. Each PCR set four replicated times. The ubiquitin (UBQ) gene was used as an internal control for data normalization. The 2^−∆∆Ct^ method was used calculating gene expression levels. The primers used in this experiment are provided in Table S15.

## Supplementary Information

**Additional file 1: Figure S1.** Quantile-quantile (Q-Q) and Manhattan plots from the genome-wide association study (GWAS) results for bacterial leaf blight (BLB) resistance used 240 indica rice varieties. (A, B, C) GWAS for BLB resistance in (A) P3_2018, (B) P3_2019 and (C) Best linear unbiased prediction (BLUP) values of P3 at two years. (D, E, F) GWAS for BLB resistance in (D) P6_2018, (E) P6_2019 and (F) Best linear unbiased prediction (BLUP) values of P6 at two years. The x-axis shows the single nucleotide polymorphism (SNPs) along each chromosome; the y-axis is the –log10P for the association.

**Additional file 2: Table S1.** Names, origin, and population structure of 259 rice accessions.

**Additional file 3: Table S2.** Sequence information on the genomes of 259 rice accessions.

**Additional file 4: Table S3.** Results of filtered SNP annotation.

**Additional file 5: Table S4.** Number of SNPs in 259 rice accessiongs using the sequenced data mapped to the Nipponbare refrence genome. MAF: minor allele frequency.

**Additional file 6: Table S5.** GWAS of P3 (BULP) resiatance with 259 rice lines using the SNP-set generated by mapping reads to the Nipponbare.

**Additional file 7: Table S6.** GWAS of P3 (2018) resiatance with 259 rice lines using the SNP-set generated by mapping reads to the Nipponbare.

**Additional file 8: Table S7.** GWAS of P3 (2019) resiatance with 259 rice lines using the SNP-set generated by mapping reads to the Nipponbare.

**Additional file 9: Table S8.** GWAS of P6 (BULP) resiatance with 259 rice lines using the SNP-set generated by mapping reads to the Nipponbare.

**Additional file 10: Table S9.** GWAS of P6 (2018) resiatance with 259 rice lines using the SNP-set generated by mapping reads to the Nipponbare.

**Additional file 11: Table S10.** GWAS of P6 (2019) resiatance with 259 rice lines using the SNP-set generated by mapping reads to the Nipponbare.

**Additional file 12: Table S11.** Statistics of Illumina transcriptome sequencing data.

**Additional file 13: Table S12.** The list of DEGs in resistant line NSIC RC154 at different inoculation times.

**Additional file 14: Table S13.** The list of DEGs in susceptible line CT 9737-6-1-3P-M at different inoculation times.

**Additional file 15: Table S14.** The 2817 DEGs were uniquely detected in the resistance line NSIC RC154.

**Additional file 16: Table S15.** Specific primers of candidate gene sequences for qRT-PCR.

## Data Availability

Sequencing data of rice were deposited at the National Center for Biotechnology (NCBI) Sequence Read Archive (SRA) under bioproject SRR11747259-11,747,279 (for RNA-Seq) and bioproject PRJNA598020 (for genome re-sequencing).

## Abbreviations

BLB: Bacterial leaf blight
Xoo: Xanthomonas Oryzae pv. Oryzae
SNPs: Single nucleotide polymorphisms
GWAS: Genome-wide association study
QTL: Quantitative trait loci
LD: Linkage disequilibrium
CDS: Coding sequences
PCA: Principal component analysis
DSI: Disease severity index
ANOVA: Analysis of variance
MLM: Mixed linear model
BLUP: Best linear unbiased prediction
DEGs: Differentially expressed genes
hpi: H post-inoculation
GO: Gene ontology
KEGG: Kyoto Encyclopedia of Genes and Genomes
ROS: Reactive oxygen species
NJ: Neighbor-joining
FDR: False discovery rate
FPKM: Fragments per kilobase of transcript sequence per million

## References

[1] Chen W, Gao Y, Xie W, Gong L, Lu K, Wang WS (2014). Genome-wide association analyses provide genetic and biochemical insights into natural variation in rice metabolism. Nat Genet.

[2] Nino-Liu DO, Ronald PC, Bogdanove AJ (2010). Xanthomonas oryzae pathovars: model pathogens of a model crop. Mol Plant Pathol.

[3] Khush GS, Mackill DJ, Sidhu GS, Banta SJ (1989). Breeding rice for resistance to bacterial blight. Bacterial blight of rice.

[4] Zhang Q (1991). Genetic evaluation and utilization of resistance to rice bacterial blight in China. Sci Agric Sin.

[5] Saha S, Garg R, Biswas A, Rai A (2015). Bacterial diseasesof rice: an overview. J Pure Appl Microbiol.

[6] Srinivasan B, Gnanamanickam SS (2005). Identification of a new source of resistance in wild rice, Oryza rufipogon to bacterial blight of rice caused by Indian strains of Xanthomonas oryzae pv. oryzae. Curr Sci.

[7] Ogawa T (1993). Methods and strategy for monitoring race distribution and identification of resistance genes to bacterial leaf blight (Xanthomonas campetris pv. oryzae) in rice. Jpn Agric Res Q.

[8] Lee KS, Rasabandith S, Angeles ER, Khush GS (2003). Inheritance of resistance to bacterial blight in 21 cultivars of rice. Phytopathology.

[9] Busungu C, Taura S, Sakagami J-I, Ichitani K (2016). Identification and linkage analysis of a new rice bacterial blight resistance gene from XM14, a mutant line from IR24. Breed Sci.

[10] Chen S, Huang Z, Zeng L, Yang J, Liu Q, Zhu X (2008). High-resolution mapping and gene prediction of Xanthomonas Oryzae pv. Oryzae resistance gene Xa7. Mol Breed.

[11] Jiang GH, Xia ZH, Zhou YL, Wan J, Li DY, Chen RS (2006). Testifying the rice bacterial blight resistance gene xa5 by genetic complementation and further analyzing xa5 (Xa5) in comparison with its homolog TFIIA1. Mol Genet Genomics.

[12] Song WY, Wang GL, Chen LL, Kim HS, Pi LY, Holsten T (1995). A receptor kinase-like protein encoded by the rice disease resistance gene, Xa21. Science (80-).

[13] Tian DS, Wang JX, Zeng X, Gu KY, Qiu CX, Yang XB (2016). The rice TAL effector-dependent resistance protein XA10 triggers cell death and calcium depletion in the endoplasmic reticulum. Plant Cell.

[14] Sun XL, Cao YL, Yang ZF, Xu CG, Li XH, Wang SP (2004). Xa26, a gene conferring resistance to Xanthomonas oryzae pv. oryzae in rice, encodes an LRR receptor kinase-like protein. Plant J.

[15] Gu K, Yang B, Tian D, Wu L, Wang D, Sreekala C (2005). R gene expression induced by a type-III effector triggers disease resistance in rice. Nature.

[16] Chu ZH, Fu BY, Yang H, Xu CG, Li ZK, Sanchez A (2006). Targeting xa13, a recessive gene for bacterial blight resistance in rice. Theor Appl Genet.

[17] Zhang F, Zhuo DL, Zhang F, Huang LY, Wang WS, Xu JL (2014). Xa39, a novel dominant gene conferring broad-spectrum resistance to Xanthomonas oryzae pv. oryzae in rice. Plant Pathol.

[18] Kim SM, Suh JP, Qin Y, Noh TH, Reinke RF, Jena KK (2015). Identification and fine-mapping of a new resistance gene, Xa40, conferring resistance to bacterial blight races in rice (Oryza sativa L.). Theor Appl Genet.

[19] Liang LQ, Wang CY, Zeng LX, Wang WJ, Feng JQ, Chen B (2017). The rice cultivar Baixiangzhan harbours a recessive gene xa42 (t) determining resistance against Xanthomonas oryzae pv. oryzae. Plant Breed.

[20] Liu J, Wang X, Mitchell T, Hu Y, Liu X, Dai L (2010). Recent progress and understanding of the molecular mechanisms of the rice–Magnaporthe oryzae interaction. Mol Plant Pathol.

[21] Bandillo N, Raghavan C, Muyco PA, Sevilla MAL, Lobina IT, Dilla-Ermita CJ (2013). Multi-parent advanced generation inter-cross (MAGIC) populations in rice: progress and potential for genetics research and breeding. Rice.

[22] Chen S, Liu X, Zeng L, Ouyang D, Yang J, Zhu X (2011). Genetic analysisand molecular mapping of a novel recessive gene xa34(t) for resistance against Xanthomonas oryzae pv. oryzae. Theor Appl Genet.

[23] Vikal Y, Bhatia D. Genetics and genomics of bacterial blight resistance in rice. In: Advances in international rice research. Jinquan Li, IntechOpen; 2017. p. 175–213.

[24] Burghardt LT, Young ND, Tiffin P (2017). A guide to genome-wide association mapping in plants: genome-wide association mapping in plants. Curr Protoc Plant Biol.

[25] Lipka AE, Tian F, Wang Q, Peiffer J, Li M, Bradbury PJ (2012). GAPIT: genome association and prediction integrated tool. Bioinformatics.

[26] Li TG, Ma XF, Li NY, Zhou L, Liu Z, Han HY (2017). Genome-wide association study discovered candidate genes of Verticillium wilt resistance in upland cotton (Gossypium hirsutum L.). Plant Biotechnol J.

[27] Liu MH, Kang HX, Xu YC, Peng Y, Wang D, Gao LJ, et al. Genome-wide association study identifies an NLR gene that confers partial resistance to Magnaporthe oryzae in rice. Plant Biotechnol J. 2020;18(6):1376–83. 10.1111/pbi.13300 PMC7206997 31742855

[28] Wen Z, Tan R, Zhang S, Collins PJ, Yuan J, Du W (2018). Integrating GWAS and gene expression data for functional characterization of resistance to white mould in soya bean. Plant Biotechnol J.

[29] Huang XH, Wei XH, Sang T, Zhao Q, Feng Q, Zhao Y (2010). Genome-wide association studies of 14 agronomic traits in rice landraces. Nat Genet.

[30] Liu QS, Yuan M, Zhou Y, Li XH, Xiao JH, Wang SP (2011). A paralog of the MtN3/saliva family recessively confers race-specific resistance to Xanthomonas oryzae in rice. Plant Cell Environ.

[31] Rohini G, Shalu J, Akhilesh KT, Mukesh J (2010). Genome-wide survey and expression analysis suggest diverse roles of glutaredoxin gene family members during development and response to various stimuli in rice. DNA Res.

[32] Ogo Y, Kobayashi T, Nakanishi IR, Nakanishi H, Kakei Y, Takahashi M (2008). A novel NAC transcription factor, IDEF2, that recognizes the iron deficiency-responsive element 2 regulates the genes involved in iron homeostasis in plants. J Biol Chem.

[33] Kieu NP, Aznar A, Segond D, Rigault M, Simond-Côte E, Kunz C (2012). Iron deficiency affects plant defence responses and confers resistance to Dickeya dadantii and Botrytis cinerea. Mol Plant Pathol.

[34] Mani A, Sankaranarayanan K (2018). In rice plants, several metal ions like Zn^2+^, Mn^2+^, Fe^2+^, Cd^2+^ etc. has been transported via NRAMP transporter proteins. Protein J.

[35] Zhang F, Wu ZC, Wang MM, Zhang F, Dingkuhn M, Xu JL (2017). Genome-wide association analysis identifies resistance loci for bacterial blight in a diverse collection of indica rice germplasm. PLoS One.

[36] Li N, Lin B, Wang H, Li XM, Yang FF, Ding XH (2019). Natural variation in ZmFBL41 confers banded leaf and sheath blight resistance in maize. Nat Genet.

[37] Chen HL, Wang SP, Zhang QF (2002). New gene for bacterial blight resistance in rice located on chromosome 12 identified from Minghui 63, an elite restorer line. Phytopathology.

[38] Park CJ, Ronald PC (2012). Cleavage and nuclear localization of the rice XA21 immune receptor. Nat Commun.

[39] Zhou YL, Uzokwe VNE, Zhang CH, Cheng LR, Wang L, Chen K (2011). Improvement of bacterial blight resistance of hybrid rice in China using the Xa23 gene derived from wild rice (Oryza rufipogon). Crop Prot.

[40] Korinsak S, Sriprakhon S, Sirithanya P, Jairin J, Korinsak S, Vanavichit A, et al. Identification of microsatellite markers (SSR) linked to a new bacterial blight resistance gene xa33(t) in rice cultivar ‘Ba7.’ Maejo Int J Sci Technol. 2009;3(2):235–47.

[41] Antony G, Zhou JH, Huang S, Li T, Liu B, White F (2010). Rice xa13 recessive resistance to bacterial blight is defeated by induction of the disease susceptibility gene Os-11N3. Plant Cell.

[42] Shadle GL, Wesley SV, Korth K, Chen F, Lamb C, Dixon R (2003). Phenylpropanoid compounds and disease resistance in transgenic tobacco with altered expression of L-phenylalanine ammonia-lyase. Phytochemistry.

[43] Li WQ, Shao M, Yang J, Zhong WG, Okada K, Yamane H (2013). Oscyp71Z2 involves diterpenoid phytoalexin biosynthesis that contributes to bacterial blight resistance in rice. Plant Sci.

[44] Li HJ, Li XH, Xiao JH, Wing RA, Wang SP (2012). Ortholog alleles at Xa3/Xa26 locus confer conserved race-specific resistance against Xanthomonas oryzae in rice. Mol Plant.

[45] Cao YL, Duan L, Li HJ, Sun XL, Zhao Y, Xu CG (2007). Functional analysis of Xa3/Xa26 family members in rice resistance to Xanthomonas oryzae pv. oryzae. Theor Appl Genet.

[46] Yoshimura S, Yamanouchi U, Katayose Y, Toki S, Wang ZX, Kono I (1998). Expression of Xa1, a bacterial blight-resistance gene in rice, is induced by bacterial inoculation. Proc Natl Acad Sci.

[47] Baggs E, Dagdas G, Krasileva KV (2017). NLR diversity, helpers and integrated domains: making sense of the NLR IDentity. Curr Opin Plant Biol.

[48] Cesari S, Bernoux M, Moncuquet P, Kroj T, Dodds PN (2014). A novel conserved mechanism for plant NLR protein pairs: the “integrated decoy” hypothesis. Front Plant Sci.

[49] Wu CH, Krasileva KV, Banfield MJ, Terauchi R, Kamoun S (2015). The, “sensor domains” of plant NLR proteins: more than decoys. Front Plant Sci.

[50] Kauffman HE, Reddy APK, Hsieh SPY, Merca SD (1973). A improved technique for evaluation of resistance of rice varieties to Xanthomonas oryzea. Plant Dis Rep.

[51] Uzunova M, Ecke W, Weissleder K, Röbbelen G (1995). Mapping the genome of rapeseed (Brassica napus L.). I. Construction of an RFLP linkage map and localization of QTLs for seed glucosinolate content. Theor Appl Genet.

[52] Li H, Durbin R (2010). Fast and accurate long-read alignment with Burrows-Wheeler transform. Bioinformatics.

[53] McKenna A, Hanna M, Banks E, Sivachenko A, Cibulskis K, Kernytsky A (2010). The genome analysis Toolkit: a MapReduce framework for analyzing next-generation DNA sequencing data. Genome Res.

[54] Vilella AJ, Severin J, Ureta-Vidal A, Heng L, Durbin R, Birney E (2009). EnsemblCompara GeneTrees: complete, duplication-aware phylogenetic trees in vertebrates. Genome Res.

[55] Alexander DH, Novembre J, Lange K (2009). Fast model-based estimation of ancestry in unrelated individuals. Genome Res.

[56] Yang J, Le SH, Goddard ME, Visscher PM (2011). GCTA: a tool for genome-wide complex trait analysis. Am J Hum Genet.

[57] Zhang C, Dong SS, Xu JY, He WM, Yang TL (2019). PopLDdecay: a fast and effective tool for linkage disequilibrium decay analysis based on variant call format files. Bioinformatics.

[58] Poland JA, Bradbury PJ, Buckler ES, Nelson RJ (2011). Genome-wide nested association mapping of quantitative resistance to northern leaf blight in maize. Proc Natl Acad Sci USA.

[59] Kang HM, Sul JH, Service SK, Zaitlen NA, Kong S, Freimer NB (2010). Variance component model to account for sample structure in genome-wide association studies. Nat Genet.

[60] Kim D, Pertea G, Trapnell C, Pimentel H, Kelley R, Salzberg SL (2013). TopHat 2: accurate alignment of transcriptomes in the presence of insertions, deletions and gene fusions. Genome Biol.

[61] Langmead B, Trapnell C, Pop M, Salzberg SL (2009). Ultrafast and memory-efficient alignment of short DNA sequences to the human genome. Genome Biol.

[62] Trapnell C, Williams BA, Pertea G, Mortazavi A, Kwan G, van Baren MJ (2010). Transcript assembly and quantification by RNA-Seq reveals unannotated transcripts and isoform switching during cell differentiation. Nat Biotechnol.

[63] Anders S, Huber W (2010). Differential expression analysis for sequence count data. Genome Biol.

[64] Anders S, Huber W (2012). Differential expression of RNA-Seq data at the gene level-the DESeq package.

[65] Benjamini Y, Hochberg Y (1995). Controlling the false discovery rate: a practical and powerful approach to multiple testing. J R Stat Soc Series B Methodol.

[66] Gabriel SB, Schaffner SF, Nguyen H, Moore JM, Roy J, Blumenstiel B (2002). The structure of haplotype blocks in the human genome. Science.

[67] Barrett JC, Fry B, Maller J, Daly MJ (2005). Haploview: analysis and visualization of LD and haplotype maps. Bioinformatics.

