# Supplementary material for: Genome-wide association study and transcriptome analysis discover new genes for bacterial leaf blight resistance in rice (Oryza sativa L.)
Source: BMC Plant Biol. 2021 Jun 3;21:255. doi: 10.1186/s12870-021-03041-2 (PMC8173721; doi:10.1186/s12870-021-03041-2)
Supplement: Supplementary file 1 — Additional file 1: Figure S1. Quantile-quantile (Q-Q) and Manhattan plots from the genome-wide association study (GWAS) results for bacterial leaf blight (BLB) resistance used 240 indica rice varieties. (A, B, C) GWAS for BLB resistance in (A) P3_2018, (B) P3_2019 and (C) Best linear unbiased prediction (BLUP) values of P3 at two years. (D, E, F) GWAS for BLB resistance in (D) P6_2018, (E) P6_2019 and (F) Best linear unbiased prediction (BLUP) values of P6 at two years. The x-axis shows the single nucleotide polymorphism (SNPs) along each chromosome; the y-axis is the –log10P for the association. [file 12870_2021_3041_MOESM1_ESM.docx]

**Genome-wide association study and transcriptome analysis discover new genes for bacterial leaf blight resistance in rice (*Oryza sativa* L.)**

Xinyue Shu^1,3,4#^, Aijun Wang^1,4#^, Bo Jiang^2#^, Yuqi Jiang^1,3,4^, Xing Xiang^1,3,4^, Xiaoqun Yi^1^, Shuangcheng Li^3^, Qiming Deng^3^, Shiquan Wang^3^, Jun Zhu^3^, Yueyang Liang^3^, Huainian Liu^3^, Ting Zou^3^, Lingxia Wang^3^, Ping Li^3^, Aiping Zheng^1,4^*

^1^College of Agronomy, Sichuan Agricultural University, Chengdu, China

^2^College of Life Science and Technology, Yangtz Normal University, Chongqing, China

^3^Rice Research Institute of Sichuan Agricultural University, Chengdu, China

^4^State Key Laboratory of Crop Gene Exploration and Utilization in Southwest China, Chengdu, China

# These authors contributed equally to this work.

*Author to whom correspondence should be addressed:

Prof Aiping Zheng, aipingzh@163.com

**Supplementary Figures**

**A**


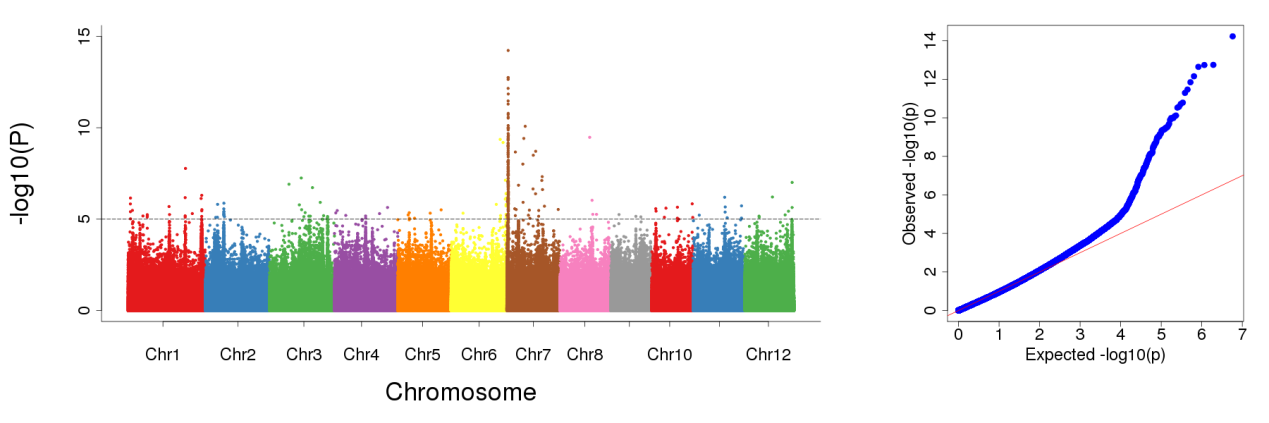


**B**


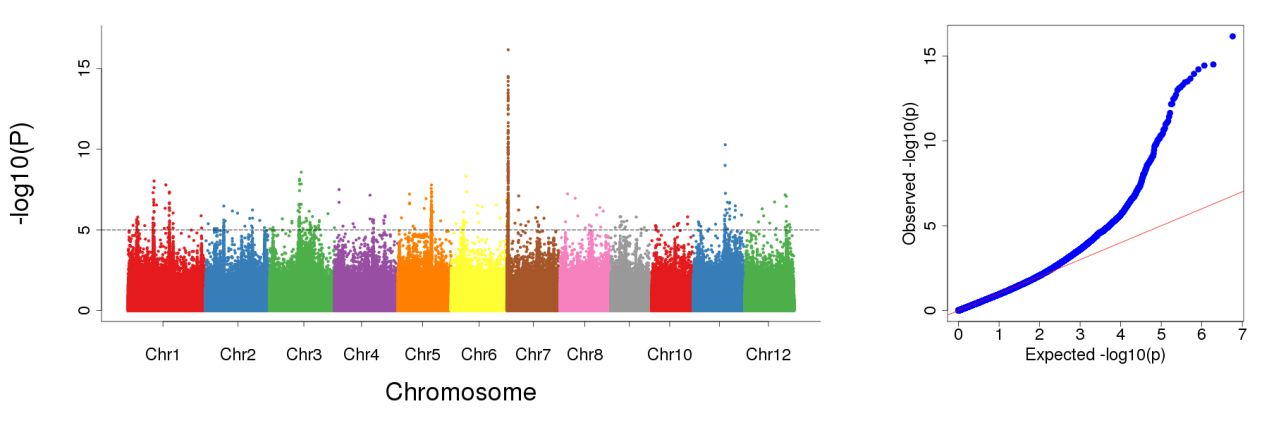


**C**


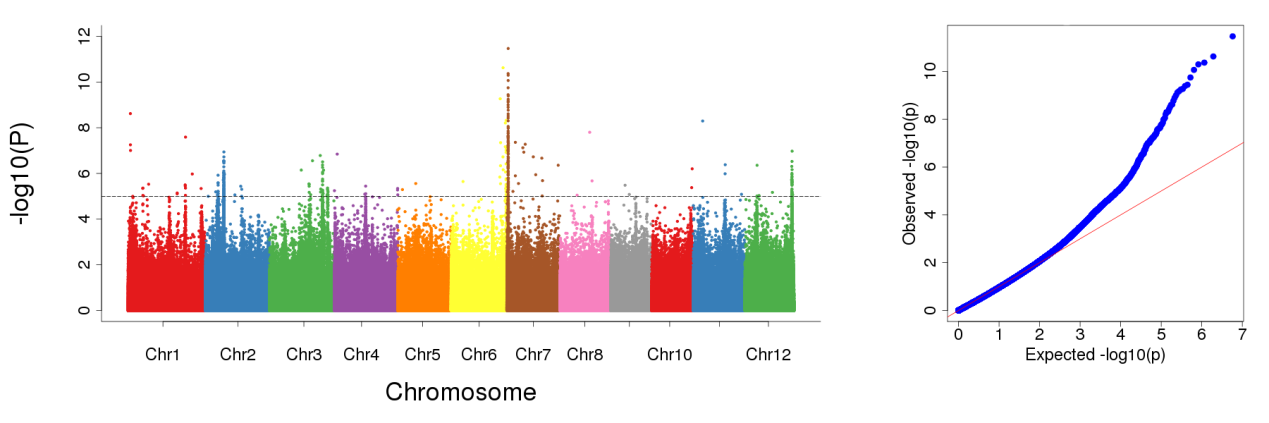


**D**


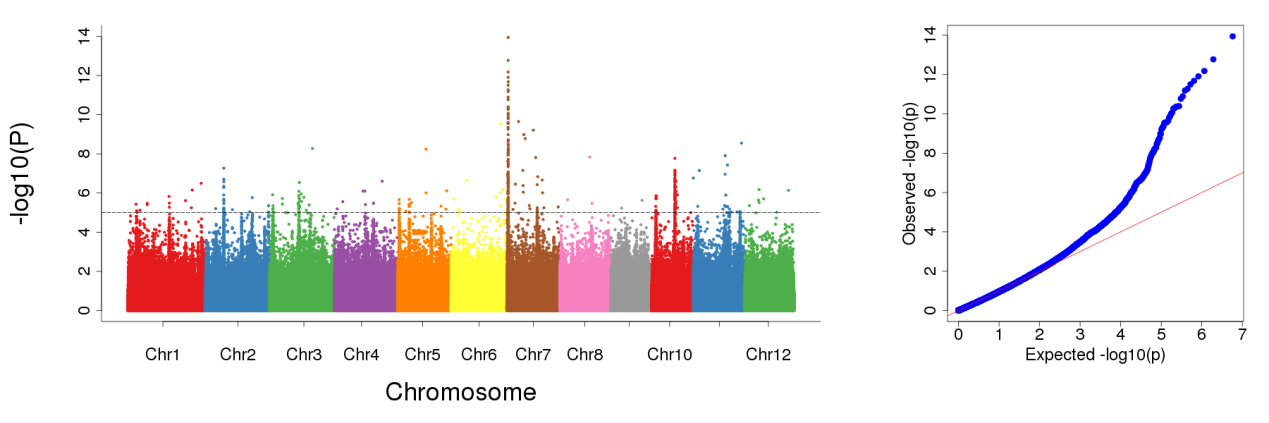


**E**


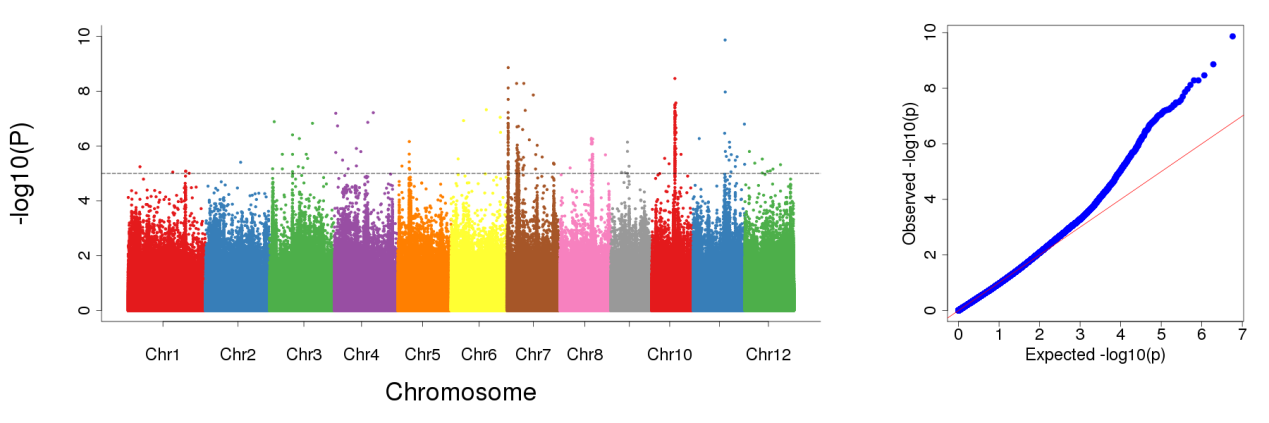


**F**


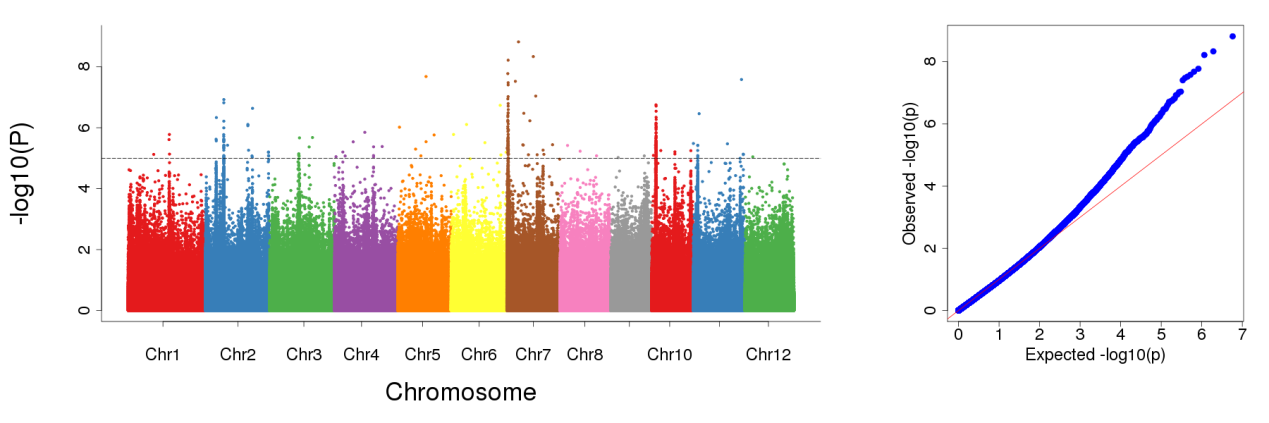


**Figure S1 Quantile-quantile (Q-Q) and Manhattan plots from the genome-wide association study (GWAS) results for bacterial leaf blight (BLB) resistance used 240 indica rice varieties.** (A, B, C) GWAS for BLB resistance in (A) P3_2018, (B) P3_2019 and (C) Best linear unbiased prediction (BLUP) values of P3 at two years. (D, E, F) GWAS for BLB resistance in (D) P6_2018, (E) P6_2019 and (F) Best linear unbiased prediction (BLUP) values of P6 at two years. The x-axis shows the single nucleotide polymorphism (SNPs) along each chromosome; the y-axis is the –log_10_P for the association.
